# Mental Health of Nursing Students: A Bibliometric Review Based on CiteSpace Visual Analysis

**DOI:** 10.1155/jonm/2169094

**Published:** 2025-03-12

**Authors:** Hong Xie, Bingyao Kang

**Affiliations:** ^1^Department of Pediatric Outpatient Nursing, West China Second University Hospital, Sichuan University, Chengdu, China; ^2^Key Laboratory of Birth Defects and Related Diseases of Women and Children Sichuan University, Ministry of Education, Chengdu, China; ^3^West China Nursing School, Sichuan University, Chengdu, China

**Keywords:** bibliometric literature, mental health, nursing students, review

## Abstract

**Background:** The mental health of nursing students directly affects their future performance, quality of patient care, and personal development. Therefore, an in-depth understanding of research in this area can assist in implementing effective measures to improve the mental health of nursing students.

**Aim:** This study used bibliometric analyses to identify and analyze articles, authors, journals, and research institutes investigating nursing students' mental health in terms of thematic structure and topic evolution, aiming to provide direction and guidance for future research.

**Methods:** In this retrospective bibliometric analysis, data were downloaded from the Web of Science Core Collection on August 15, 2024. Subsequently, CiteSpace software was employed to analyze the annual number of publications and citations, explore relationships between authors, institutions, countries, and keywords, and summarize research hotspots and frontiers in the field of nursing students' mental health.

**Results:** Articles published from database inception to August 15, 2024, were screened, leading to the retrieval of 3803 relevant articles. The United States of America was the leading country in terms of research output on the mental health of nursing students, with 982 (25.79%) published articles, while the United States Department of Health and Human Services provided the most funding. Furthermore, Happell, B was the most productive author in this field, with 80 published papers. Lastly, Nurse Education Today was the most prolific journal in nursing education.

**Conclusion:** The main recent research trends include the psychological profile of nursing students, intervention strategies for improving the mental health of nursing students, and the influence of educational methods, clinical experience, and humanistic care on the mental health of nursing students. These trends imply that researchers should develop targeted training programs, apply information technology, and implement personalized teaching to enhance the psychological resilience of the nursing student population. Moreover, higher education institutions should provide nursing students with more comprehensive and effective mental health support by maintaining mental health files, strengthening mental health education, providing psychological counseling services, and establishing social support systems.

## 1. Introduction

The American Psychological Association defines mental health as a state promoting emotional well-being, behavioral adjustment, freedom from anxiety and disabling symptoms, and the ability to build constructive relationships and manage life's demands [1]. Compared to students in other disciplines, nursing students face significantly higher levels of stress, anxiety, and sleep disorders, attributable to the academic and clinical demands of their training [2]. Healthcare workers have to take care of people with psychosis, and nurses are often at the frontline of mental healthcare [3]. Nursing students may come into contact with patients with psychosis during their studies and internships, thereby facing the risk of occupational exposure [4]. These issues not only affect the physical and mental health of nursing students, which can also constitute a series of factors that easily lead to suicide, but also endanger the safety of patients and nursing in a hospital environment [5, 6]. Consequently, nursing educators and researchers have increasingly focused on addressing the mental health concerns affecting nursing students.

Some scholars have explored the external learning and practicum environments and internal negative psychological problems, including stress, anxiety, and depression, aiming to understand the mental health of nursing students [7, 8]. Moreover, several intervention strategies have been proposed for promoting the mental health of nursing students. These measures include mindfulness interventions [9], laughter yoga [10], educational strategies [11], and resilience [12] and clinical simulation training [13]. Although different institutions and countries place varying emphasis on the mental health of nursing students, the impact of nursing students' mental health on the care quality and safety of patients has been established. Hence, nursing leaders and faculty should pay attention to the mental health of nursing students.

The number of publications on the mental health of nursing students is increasing over time. Bibliometric analysis has now become a viable option for researchers and medical practitioners. This literature analysis method can also be conducted by journal readers through built-in tools on bibliographic platforms or open-source bibliometric software. Nevertheless, a comprehensive analysis of recent publications is still required to rapidly identify the prevailing trends in research on the mental health of nursing students. In this context, bibliometric analysis may contribute to improving nursing students' mental health and provide a foundation for decision making by nursing educators and administrators. High-quality journals and high-impact papers with international influence may serve as key indicators of global trends in nursing students' mental health. Therefore, the characteristics of different publications and high-impact research papers should be analyzed to objectively reflect the current research priorities and key areas of mental health research in the nursing student population.

Bibliometric analysis is a quantitative method for analyzing literature and processing relevant data. This statistical technique employs algorithms, arithmetic, and statistics to examine, organize, and assess large amounts of data [14]. Bibliometrics applies quantitative techniques to enable scholars to objectively evaluate a substantial corpus of literature, which may comprise hundreds or more publications [15]. A meta-analysis is comparable to a bibliometric analysis in terms of its capability to assess a substantial volume of literature. In contrast, a systematic literature review focuses on a relatively limited number of sources. In this regard, systematic literature reviews are better suited for synthesizing and summarizing findings from existing literature on a specific research topic or field. However, they are primarily qualitative and may have certain biases [16]. Bibliometric and meta-analyses involve quantitative analysis methods that allow researchers to avoid or mitigate biases. Although meta-analysis and bibliometric analysis are inherently quantitative, meta-analysis is often employed as a theory extension tool to elucidate results without delving into the research process. In contrast, bibliometric analysis summarizes the bibliometric and intelligence structure of a particular field by assessing the social and structural relationships among different research components (e.g., authors, countries, institutions, and topics) [15]. Thus, all three analytical approaches described above are mutually reinforcing. However, this study leverages the strengths of bibliometric analysis to achieve the following primary aims: (1) to objectively identify thematic knowledge clusters; (2) to clarify jurisprudential networks; (3) to map social patterns; (4) to track evolutionary nuances; and (5) to identify knowledge gaps [17].

The number of bibliometric analysis studies on medicine [18] and nursing [19] has notably increased. One such bibliometric study of nursing education revealed that various domains within nursing research, including mobile learning [20], virtual simulation [21], and clinical learning environments [22], have been extensively investigated through bibliometric analysis. For example, a bibliometric analysis by Chang et al. [23] identified that workplace mental health, simulation learning, psychiatric nursing, and medication management were primary research themes in nursing education. Another bibliometric study by Readi and Mukhlis [24] on student mental health concluded that student mental health was associated with anxiety, stress, forgiveness, ethnicity, and academic engagement. The surge in research on student mental health has also attracted the attention of our research team.

Despite the increasing focus on the mental health of nursing students, bibliometric analysis in this field remains relatively scarce. This research gap may be attributed to the lack of in-depth exploration of the dynamic changes and personalized intervention strategies in the existing relevant literature. Considering these limitations, the research question should focus on utilizing bibliometric methods to comprehensively reveal the current status, trends, and challenges of the research, with the goal of providing a scientific basis for formulating targeted intervention measures. Therefore, this study employed bibliometric analysis methods to identify and investigate the most relevant articles, authors, journals, and research institutions in the field of nursing students' mental health, as well as explore the thematic structure and topic evolution, aiming to offer valuable insights and guidance for future research and practice.

## 2. Method

### 2.1. Study Design and Ethics

This retrospective bibliometric analysis primarily examined published articles and did not involve any human clinical trial studies. Consequently, ethical approval was not required from the relevant ethics review committee.

### 2.2. Data Sources

All analyzed data were downloaded from the Web of Science Core Collection (WoSCC) on August 15, 2024, amounting to 4102 retrieved articles. The WoSCC is a widespread and prominent citation database utilized to index scientific knowledge and technology as well as an efficient source of data retrieval for scientific metrological analysis. The retrieval strategy employs topic retrieval and Boolean logical operators to formulate retrieval expressions. These expressions are as follows: (TS = (“mental health” OR “mental disorder” OR “mental illness” OR wellbeing OR suicide OR psychosis OR anxiety OR depression OR “mental hygiene” OR “well-being”) AND TS = (“nursing student∗” OR “student nurs∗” OR “nursing undergraduat∗” OR “undergraduate nurs∗” OR “nursing education”)).

### 2.3. Study Inclusion/Exclusion Criteria

Study inclusion criteria were literature related to the mental health of nursing students, original research or review type of articles, and English language publications. Study exclusion criteria included literature such as meeting abstracts, editorial material, letters, news items, and duplicates. Two researchers read the titles and abstracts independently and removed literature not related to the mental health of nursing students. After the screening process, 3803 publications were finally included in the analysis, comprising 3513 original research studies and 290 reviews.

### 2.4. Analytical Methods

**Figure 1:**
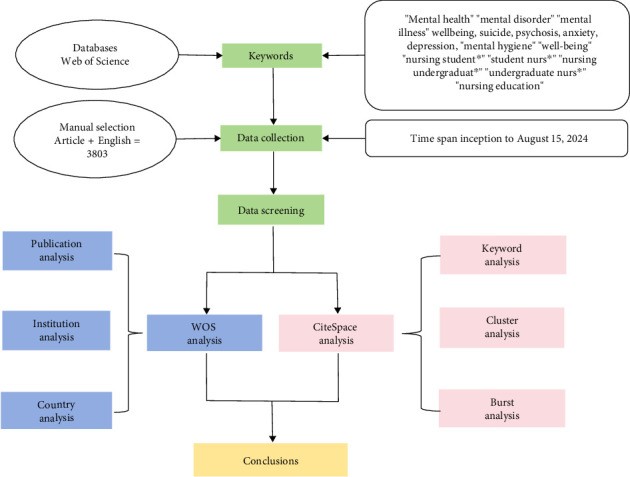
The flowchart depicting the procedures carried out in the bibliometric review.

The bibliometric analysis process conducted in this study is illustrated in Figure 1. CiteSpace was employed to analyze the annual number of publications and citations, explore the relationships between authors, institutions, countries, and keywords, and identify the emerging trends and research frontiers in the field of nursing students' mental health. Furthermore, keywords that exhibited a notable increase in citations over time were detected in this analysis. CiteSpace knowledge graphs include a variety of nodes and links. The identification of hot spots or turning points in this domain was typically accomplished by distinguishing nodes with high centrality. First, the literature retrieved from WoSCC, including the cited references, was downloaded and exported in RefWorks format. The corresponding data were then exported in text format to establish a dataset. Subsequently, duplicate literature was excluded, and the remaining literature was visualized using CiteSpace 6.1.R6 software. The selected period for the bibliometric and visual analysis was from 1928 to 2024. A cluster analysis using CiteSpace was conducted to determine the principal topics based on keyword co-occurrence. The obtained clusters were then assessed using the silhouette function. In this analysis, a partition structure was deemed significant if the modularity value (*Q*) exceeded 0.3, while the clustering results were considered significant if the silhouette value (*S*) was > 0.7 [25].

## 3. Results

### 3.1. Bibliometric Analysis of Publication Years

**Figure 2:**
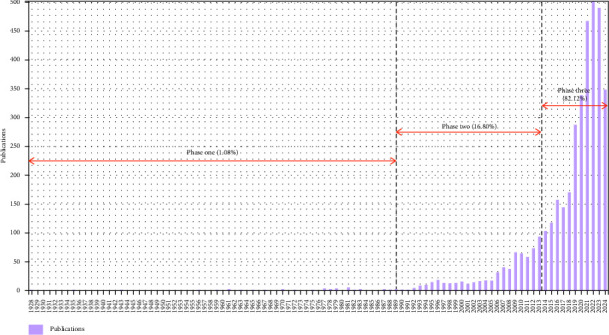
Year-wise publication of bibliometric papers from inception to August 15, 2024.

In this study, a comprehensive collection of articles examining the mental health of nursing students was achieved, spanning the period from 1928 to 2024. As depicted in Figure 2, the annual publication statistics demonstrated that the number of publications in this field has notably increased over time. Additionally, the number of publications exhibited three discernible phases. In the initial phase, which spanned 64 years from 1928 to 1992, only 1.08% of the total number of articles was published, with no more than five published in any given year. During the second phase or developmental period from 1993 to 2013, 16.80% of the total articles were published. Finally, the third phase or the “rapid period” (2014–2024) witnessed a considerably accelerated production of articles, where the number reached 502 by 2022. As of August 15, 2024, 348 more articles have been published, with additional publications expected in the following years.

### 3.2. Bibliometric Analysis of Countries and Institutions

Our analysis revealed that 114 countries and 3594 institutions have published research on the mental health of nursing students.  Table 1 ranks the top 10 countries and institutions with the highest number of publications on the mental health of nursing students, along with the corresponding number and percentage of articles. As demonstrated in Table 1, the United States of America was the leading country in terms of research output on the mental health of nursing students, with 982 published articles that represented 25.79% of the total. Australia was the second most prolific nation, with 460 (12.08%) articles, followed by England with 346 (9.09%) articles. Central Queensland University had the highest research output, with 70 (1.84%) published papers.

### 3.3. Bibliometric Analysis of Authors and Funding Agencies

**Figure 3:**
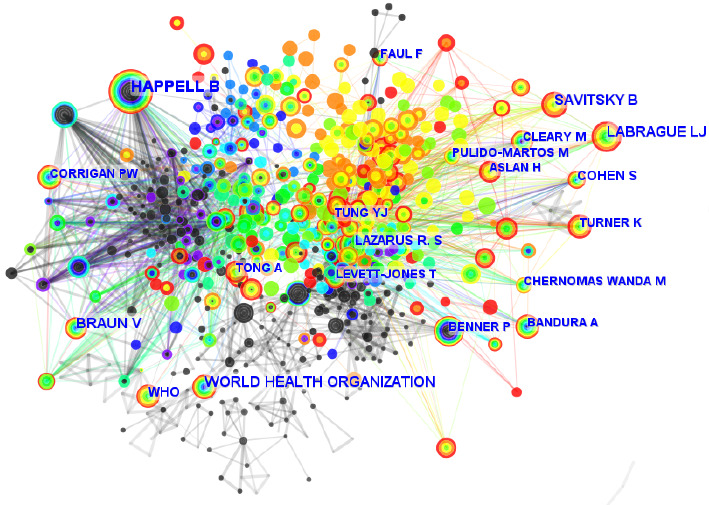
Visualization and analysis of co-authors in publications related to nursing students' mental health—CiteSpace visualization. Circular nodes represent authors of the papers, and links between the nodes indicate partnerships.

Table 2 lists the 10 most prolific authors who have published work related to nursing students' mental health. Among them, Happell, B (80) was the most productive author in this field. A co-citation graph was also constructed using the “cited authors” node type to examine author collaboration among those with publications on the mental health of nursing students (Figure 3). The author with the highest number of citations (385 citations) was the World Health Organization (WHO), followed by Happell, B with 364 citations and Labrague, LJ with 190 citations. In the co-citation graph, each circle represents a node, while the circle size is proportional to the number of articles published by a specific author. Moreover, the thickness and number of connections between the nodes indicated the collaboration level, with greater thickness and number of connections implying closer cooperation. The funding agency statistics of the literature data were also analyzed using the WoSCC statistical tool. The results showed that the 3803 publications on nursing students' mental health were funded by 1377 funding agencies, with the United States Department of Health and Human Services providing the most funding (*n* = 49, 1.29%), followed by the National Institutes of Health of the United States of America (*n* = 34, 0.89%) and the National Natural Science Foundation of China (*n* = 30, 0.79%). The top 10 funding agencies are also displayed in Table 2.

### 3.4. Bibliometric Analysis of Journals and Co-Cited References

Table 3 presents the top 10 journals and co-cited references in the field of nursing students' mental health. *Nurse Education Today* was the most productive journal (*n* = 422). The most co-cited reference was published by Savitsky et al. [26], which revealed that over 50% of Israeli nursing students experienced moderate-to-severe anxiety during the coronavirus disease 2019 (COVID-19) pandemic and subsequent lockdown. Distance teaching provided by nursing staff has been suggested as an effective means of reducing nursing students' anxiety levels. In a review of interventions for reducing stressors in nursing undergraduates, Turner and McCarthy [27] found that most approaches involved the current development or improvement of coping skills. However, further research is required to ascertain the effectiveness of such strategies. In a systematic review by Tung et al. [28], a high prevalence of depression was found among nursing students. All these findings indicate that higher education institutions should provide comprehensive stress reduction interventions and counseling services to all students, thereby contributing to reducing the alarming rates of depression within this vulnerable demographic.

### 3.5. Bibliometric Analysis of Keyword Co-Occurrence and Clustering

**Figure 4:**
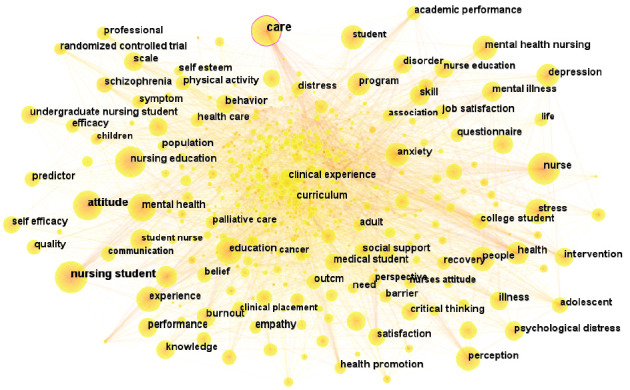
Network of main keywords in publications related to nursing students' mental health—CiteSpace visualization.

Keywords associated with the mental health of nursing students were employed to construct a co-occurrence network. This network represented the research topics related to the mental health of nursing students, with the size of each circle in the network proportional to the frequency of a particular keyword.  Table 4 presents the 10 most frequently occurring keywords. The concept of centrality is crucial in evaluating the importance of the nodes represented in the graph. Keywords with high centrality indicate the significance of related research in the field of nursing students' mental health. Furthermore, a purple circle as the outer ring of a node signifies a higher node centrality in the co-occurrence network graph.  Figure 4 illustrates that the keyword with the highest centrality was “care,” with a centrality value of 0.13.

**Figure 5:**
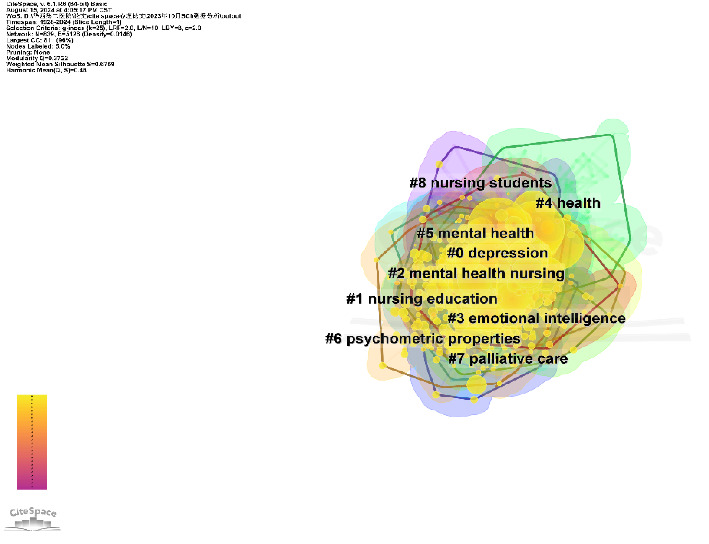
Cluster analysis of keywords in research on the mental health of nursing students—CiteSpace visualization.

A further clustering analysis of the keywords identified nine clusters, as shown in Figure 5 and Table 5. The colored blocks in Figure 5 represent the clusters, with the cluster size increasing with a smaller cluster number and a higher number of included keywords. The modularity value (*Q* = 0.3722) denoted a significant partition structure, while the silhouette value (*S* = 0.6759) corresponded to noteworthy clustering results [25].

### 3.6. Bibliometric Analysis of Keywords With Citation Bursts

**Figure 6:**
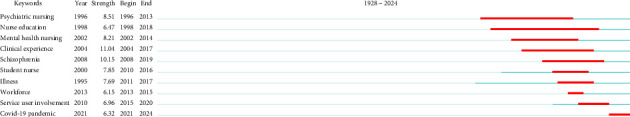
Top 10 keywords with the strongest citation bursts.

Keywords with citation bursts are words that are used frequently or occur with greater frequency over a relatively short period. The analysis of these frequency changes allows the identification of frontiers and trends within a research field.  Figure 6 depicts the 10 most prevalent keywords with citation bursts in the period from 1928 to 2024. The keywords are “psychiatric nursing,” “nurse education,” “mental health nursing,” “clinical experience,” “schizophrenia,” “student nurse,” “illness,” “workforce,” “service user involvement,” and “covid-19 pandemic.” The year indicates the initial year of the corresponding keyword's appearance. Additionally, the beginning and end of the keyword's appearance with citation bursts are indicated by the start and end years, respectively. Lastly, the length of the red line denotes the duration of the keyword burst.

## 4. Discussion

### 4.1. General Characteristics

This study represents the first analysis of the available literature on nursing students' mental health utilizing CiteSpace software. A total of 3803 papers were retrieved from the WoSCC from database construction to August 15, 2024, with the number of publications increasing every year. This outcome indicates that nursing students' mental health is an active research field that has received extensive attention from researchers. A total of 114 countries have published articles pertinent to this field, among which the United States of America accounts for the highest number of papers (*n* = 982, 25.79%), underlining its dominance in this field. Australia and England were the next two most prolific contributors.

Mental health services in the United States of America are relatively widespread, and mental health issues receive a high level of societal interest [29]. As evidenced in Table 2, the United States of America Department of Health and Human Services is the primary funding source in this research area. The United States of America has a wealth of research resources and financial support, which provides optimal conditions and assurances for research on nursing students' mental health. These advantageous conditions enable in-depth exploration and positive outcomes in related research, further underscoring the prominent position of the United States of America in this field. The level of attention, resource inputs, policy support, and education systems for supporting the mental health of nursing students vary across different countries. These differences may result in nursing students receiving distinct levels of support and assistance when experiencing mental health problems. Therefore, cooperation and exchanges between countries and the joint exploration of effective models of support and services should be strengthened to improve the mental health of nursing students globally. Countries should also develop policies and measures tailored to their specific cultural, economic, and political contexts to alleviate the mental health of this population.

Our table displays the distribution of research institutions involved in this field, which may assist researchers in identifying and selecting relevant institutions with which they can collaborate. As demonstrated in Table 1, higher education academic institutions were the research institutions predominantly publishing papers on the mental health of nursing students. The top three institutions were located in Australia, the United States of America, and the United Kingdom, emphasizing that the institutions in these three countries contributed substantially in terms of absolute and relative impact. The present study found that the contributions between institutions and countries are consistent. As presented in Table 3, the three most frequently cited journals in the field of nursing education were Nurse Education Today, Nurse Education in Practice, and the Journal of Nursing Education. These international journals are at the forefront of advancing the theory and pedagogy of nursing education. All these findings suggest that the mental health of nursing students is a prominent area of focus within the nursing education field.

### 4.2. Research Topics and Emerging Trends

Keywords are used to visually express the topic of a particular paper. Keyword analysis allows researchers to rapidly understand the progress and dynamics in related fields. The results from our keyword co-occurrence and cluster analysis demonstrated that the predominant recent research trends in the mental health of nursing students included suicide risk, anxiety, depression, and negative emotions, as well as psychological interventions such as resilience training, mindfulness meditation, emotional reactions, and stress management. Moreover, research trends were identified in the education and career development of nursing students, encompassing nursing education, teaching methods, clinical experience, and career choice. Furthermore, the role of humanistic care and attitudes toward mental disorders in the context of the mental health of nursing students represents a noteworthy area of interest within the field of nursing research.

The mental health of nursing students is closely linked to psychological problems such as stress, anxiety, and depression [30]. The results of a study by Tung et al. revealed a high depression prevalence of 34% in nursing students [28]. The primary causes of stress among nursing students are related to academic (including exams, assignments, and other study-related issues) and clinical settings (e.g., fear of making mistakes in practice, heavy workload, and encounters with dying patients). Such factors may contribute to the development of depression and increase suicide risk in this population [5, 28, 31]. In particular, the COVID-19 pandemic had a detrimental impact on the mental health of nursing students [32]. Resilience training and mindfulness meditation have been demonstrated to serve as protective factors in establishing emotional regulation, enhancing nursing students' resilience and self-care, and diminishing stress, anxiety, and depression [12, 33]. Although negative emotions cannot be completely eliminated, these outcomes highlight the necessity for nursing researchers and educators to prioritize the mental health of nursing students. Additionally, these findings underscore that integrating resilience training, mindfulness meditation, and stress management programs into the Bachelor of Nursing program offers potential benefits in improving the well-being of nursing staff.

Education is a highly efficacious instrument for fostering mental health and mental healthcare [34]. The pervasive integration of information technology in nursing education, particularly during the advent of the novel COVID-19 pandemic, has precipitated a shift toward distance learning as the predominant pedagogical approach. However, nursing students exhibit a proclivity for traditional educational methodologies [35, 36]. Moreover, the traditional nursing education models have been found to be inadequate for ensuring that all nursing students can manage complex clinical problems. Nevertheless, simulation-based education has been shown to facilitate the acquisition of the knowledge, skills, and confidence required to recognize and manage mental healthcare issues [37]. Simulation education is currently trending toward computer-based or virtual simulation, with related research indicating that simulation-based learning is an effective method for aligning educational theory with clinical practice [38]. Thus, future simulation-based teaching scenarios in mental health education for nursing students are recommended to be created. However, providing technical support for simulation technology-based teaching models is remarkably challenging in developing countries. Along with teaching methods, clinical education and humanistic care are crucial aspects to nurture the mental well-being of nursing students.

Approximately 1 billion people worldwide are affected by mental disorders [39]. The stigmatizing attitude of nursing staff toward patients with mental illness is a major global challenge [40]. A substantial proportion of nursing students are reluctant to care for individuals with mental illness [41]. A majority of studies have demonstrated that providing students with the requisite educational preparation to interact with individuals with mental illness, along with clinical practice or classroom learning, can effectively reduce this stigma [42, 43]. Nurses play a pivotal role in mental healthcare, with risk assessment being a crucial aspect of this care [44]. Although nursing students may or may not practice mental healthcare in the future, they must provide mental healthcare to patients in varied settings, including primary care, inpatient mental healthcare, and specialty mental healthcare [45, 46]. Therefore, researchers should identify measures and/or policies to help decrease the stigmatization of mental health disorders among nursing students. Additionally, this population should be provided with education in risk assessment and safety planning to assist them in developing coping skills for mental healthcare.

## 5. Limitations

This study analyzes only English-language literature from the Web of Science database, which may introduce a citation bias due to the exclusion of literature from other databases and languages, potentially leading to incomplete research coverage. While the visualizations produced by CiteSpace provide an intuitive representation, they may not fully capture the complex relationships within the literature or the complete dynamics of the research. Moreover, the interpretation of data results could be influenced by the subjective judgment of the researchers. Although CiteSpace analysis utilizes multiple algorithms for constructing and analyzing citation networks, discrepancies between these algorithms may arise, affecting ability to reveal relationships among the literature. These limitations collectively impact the comprehensiveness and accuracy of the analysis results in this study. Therefore, future research endeavors should incorporate additional methodologies, such as qualitative analysis and case studies, to comprehensively assess and analyze the research trends in the domain of mental health among nursing students.

## 6. Conclusion

This study examines the trends in the volume of papers published on mental health in nursing students and assesses the extent of collaboration among countries, institutions, and authors. Recent prominent research trends include the psychological profiling of nursing students, interventions for mental health issues in this population, and the impact of educational approaches, clinical exposure, and compassionate care on their mental well-being.

To address common psychological challenges such as anxiety, depression, and stress among nursing students, targeted training programs should be developed. These programs should help students recognize and understand their psychological states, equipping them with effective coping mechanisms and skills. Moreover, fostering psychological resilience is essential for maintaining a positive attitude and emotional stability in the face of academic and job-related stressors. This can be achieved through stress management, resilience training, and emotion regulation programs. Additionally, enhancing students' professional identity is crucial. By offering specialized nursing courses, students' pride and sense of belonging within the nursing profession can be nurtured. This will help mitigate psychological issues stemming from identity crises and boost students' confidence and motivation. Improving training methods involves diversifying teaching approaches, utilizing technology, and implementing personalized instruction. Higher education institutions can enhance mental health support for nursing students by establishing mental health records, improving mental health education, offering psychological counseling services, and creating social support systems.

## Figures and Tables

**Table 1:** Top 10 countries and institutions.

Rank	Country	*N* (%)	Institution	*N* (%)
1	USA	982 (25.79)	Central Queensland University	70 (1.84)
2	Australia	460 (12.08)	University System of Ohio	53 (1.39)
3	England	346 (9.09)	University of London	49 (1.29)
4	China	307 (8.06)	University of Wollongong	49 (1.29)
5	Canada	198 (5.20)	Universidade De Sao Paulo	44 (1.16)
6	Spain	196 (5.59)	University of Newcastle	43 (1.13)
7	Turkey	184 (4.83)	Hong Kong Polytechnic University	43 (1.13)
8	South Korea	123 (3.23)	University of Texas System	43 (1.13)
9	Brazil	99 (2.60)	University of Nottingham	42 (1.10)
10	Saudi Arabia	99 (2.60)	Monash University	41 (1.08)

**Table 2:** Top 10 authors and funding agencies on mental health for nursing students.

Rank	Authors	*N* (%)	Funding agencies	*N* (%)
1	Happell, Brenda	80 (2.10)	United States Department of Health Human Services	49 (1.29)
2	Moxham, Lorna	40 (1.05)	National Institutes of Health NIH USA	34 (0.89)
3	Platania-Phung, Chris	28 (0.74)	National Natural Science Foundation of China NSFC	30 (0.79)
4	Patterson, Christopher	21 (0.55)	National Research Foundation of Korea	20 (0.53)
5	Lahti, Mari	19 (0.50)	Conselho Nacional De Desenvolvimento Cientifico E Tecnologico CNPQ	16 (0.42)
6	Horgan, Aine	17 (0.45)	Erasmus	16 (0.42)
7	MacGabhann, Liam	16 (0.42)	Ministry of Science and Technology Taiwan	15 (0.39)
8	Perlman, Dana	16 (0.42)	Spanish Government	13 (0.34)
9	Allon, Jerry	16 (0.42)	Australian Government	12 (0.32)
10	Hals, Elisabeth	16 (0.42)	Coordenacao De Aperfeicoamento De Pessoal De Nivel Superior CAPES	11 (0.29)

**Table 3:** Top 10 journals and co-cited references sorted by the number of citations.

Rank	Journals	*N* (%)	Co-cited reference	Count
1	Nurse Education Today	422 (11.10)	Savitsky et al. (2020)	140
2	Nurse Education in Practice	206 (5.42)	Turner et al. (2017)	105
3	Journal of Nursing Education	145 (3.81)	Tung et al. (2018)	93
4	International Journal of Mental Health Nursing	128 (3.37)	Happell et al. (2013)	86
5	Journal of Advanced Nursing	95 (2.50)	Aslan et al. (2021)	75
6	Journal of Psychiatric and Mental Health Nursing	95 (2.50)	Cao et al. (2020)	71
7	BMC nursing	89 (2.34)	Bartlett et al. (2016)	61
8	Clinical Simulation in Nursing	83 (2.18)	Chernomas Wanda et al. (2013)	61
9	Journal of Clinical Nursing	82 (2.16)	Reeve et al. (2013)	51
10	Journal of Professional Nursing	81 (2.13)	Li et al. (2020)	48

**Table 4:** Top 10 keyword on mental health for nursing students.

Rank	Count	Keywords	Centrality	Keywords
1	1351	Nursing student	0.13	Care
2	664	Mental health	0.08	Health
3	518	Education	0.08	Nursing student
4	518	Nursing education	0.07	Nurse
5	497	Nurse	0.06	Anxiety
6	438	Anxiety	0.06	Attitude
7	427	Stress	0.06	Behavior
8	345	Care	0.06	Disorder
9	335	Experience	0.05	Adolescent
10	326	Health	0.05	Critical thinking

**Table 5:** Keyword cluster analysis.

Label (LLR)	Silhouette	Included keywords	Mean (year)
Nursing students	0.815	Mental health; resilience training; health promotion; stress management; mindfulness meditation	2003
Palliative care	0.777	Spiritual care; end-of-life care; humanities; cross-cultural issues; covid-19 pandemic	2014
Psychometric properties	0.773	Predicting intention; lgbt; intimate partner violence; health care; nursing care	2013
Mental health	0.738	Clinical experience; career choice; medical students; attitudes towards mental illnesses; suicide risk	2011
Health	0.782	Suicide risk; stigmatizing attitude; mindfulness; workplace violence; psychiatry	2001
Emotional intelligence	0.564	Emotional reactions; physical activity; qualitative study; mental health nursing; academic burnout	2016
Mental health nursing	0.598	Stigma; psychiatric nursing; anxiety; schizophrenia; clinical placement	2008
Nursing education	0.563	Nursing education; simulation; virtual reality; standardized patient; self-confidence	2014
Depression	0.664	Mental health; negative emotions; social support; stress; psychological distress	2009

## Data Availability

All citation and bibliometric data used in this study were retrieved from the Web of Science Core Collection.
